# Berbamine attenuates hind limb ischemia-reperfusion injury by eliminating lipid ROS and inhibiting p65 nuclear translocation

**DOI:** 10.3389/fphar.2025.1509860

**Published:** 2025-03-11

**Authors:** Lei Zheng, Biao Zhao, Run Ji, Zhenxi Zhang, Yutong Liu, Xiaoqi Zhao, Jing Cai, Tong Qiao

**Affiliations:** ^1^ Department of Vascular Surgery, Nanjing Drum Tower Hospital, Affiliated Hospital of Medical School, Nanjing University, Nanjing, China; ^2^ Department of Vascular Surgery and Intervention, The Affiliated Suzhou Hospital of Nanjing Medical University, Suzhou, China; ^3^ Department of General Surgery, Nanjing Drum Tower Hospital, Affiliated Hospital of Medical School, Nanjing University, Nanjing, China

**Keywords:** hind limb ischemia reperfusion injury, lipid ROS, p65, inflammation, muscle injury, antioxidant

## Abstract

This research aims to explore whether Berbamine (BBM) can mitigate tissue damage in mice resulting from hind limb muscle ischemia-reperfusion by scavenging lipid ROS and inhibiting p65 nuclear translocation. The hind limb ischemia-reperfusion (IR) injury model in mice was employed. Forty-eight mice (n = 12 per group) were randomly allocated into four groups: Sham group, IR group, IR + BBM (20 mg/kg) group, and IR + BBM (50 mg/kg) group. We observed that BBM pretreatment shielded against muscle damage and diminished levels of cell apoptosis compared to the control group. The mechanism likely involves reducing the movement of p65 into the nucleus and lessening the build-up of lipid ROS in muscle tissue. This action helps to decrease the release of substances that cause inflammation, ultimately reducing the inflammation in tissues that occurs as a result of hind limb IR. Our findings suggest that BBM has a protective impact on hindlimb ischemia-reperfusion injury, potentially due to its capacity to eliminate tissue lipid ROS and prevent p65 nuclear translocation.

## 1 Introduction

Lower limb ischemia-reperfusion injury (LLIRI) is a complex pathophysiological process that typically occurs when blood supply is restored after prolonged ischemia in the lower limbs, such as in cases of lower limb arterial occlusion, severe trauma, blood flow interruption during surgery, or prolonged compression (Duehrkop et al., 2014; Bank and Song, 2013). The restoration of blood supply triggers a series of inflammatory responses and cellular damage. The occurrence mechanism of LLIRI involves multiple aspects, including the production of free radicals, the activation of inflammatory responses, the initiation of apoptosis, and the disruption of microcirculation (Hosszu et al., 2017; Kuroda et al., 2020). The generation and buildup of lipid Reactive oxygen species (ROS) in tissues following ischemia-reperfusion are newly identified significant contributors to tissue damage (Mendoza et al., 2024). The above pathological mechanism, where inflammation and lipid ROS mutually promote each other, further causes organ dysfunction and even death, hence there is an urgent need to find relevant treatment methods.

Treatment strategies for LLIRI primarily include the use of anti-inflammatory medications, the application of antioxidants, blood dilution to improve microcirculation, and surgical interventions (Furubeppu et al., 2021; Bihari et al., 2017). Berbamine (BBM) is a natural compound extracted from the traditional Chinese medicine *Berberis amurensis* and possesses a variety of biological activities. It has the function of inhibiting Ca^2+^/calmodulin-dependent protein kinase II γ (CaMKII γ) and p65 (Liang et al., 2009; Zhao et al., 2023; Yin et al., 2022; Gu et al., 2012; Ling et al., 2013). Additionally, the CaMKII-mediated activation of P65 caused by ischemia-reperfusion injury is a significant reason for the initiation of inflammation (Ling et al., 2013). Recent studies have found that Berbamine belongs to a class of bisbenzylisoquinoline (BBIQ) compounds, which are able to scavenge 1,1-diphenyl-2-picryl-hydrazyl (DPPH) and prevent the accumulation of lipid ROS in living cells (Fan et al., 2022). Previous studies have also found that BBM can protect the myocardium and brain from ischemia/reperfusion injury by inhibiting HMGB1 (Zheng et al., 2017; Zhu et al., 2018). Therefore, we speculate that BBM can be used to prevent hind limb IR-induced acute injury.

In our study, a classical hindlimb IR model was built in adult male C57BL/6 mice to investigate whether BBM could have a potential protective role in hindlimb IR injury and to illustrate its pathophysiological and pharmacological mechanisms.

## 2 Methods and materials

### 2.1 Animals

Adult male C57BL/6 mice, aged 8 weeks and weighing 25.0 ± 3.0 g, were provided with sterile water and a standard rodent diet. These mice were housed in sterile cages supplied by the Model Animal Research Center at Nanjing University. All procedures were approved by the Animal Investigation Ethics Committee of Nanjing University and followed the regulations of the National Institutes of Health (NIH Publication No. 85–23, revised 1996). All efforts were made to minimize animal pain and suffering throughout the studies.

### 2.2 Hindlimb ischemia–reperfusion model

The hindlimb IR injury model was established according to previous studies (Zhao et al., 2022). The mice were anesthetized (i.p.) with Tribromoethanol (Aibei M2940, Nanjing, China) 270 mg/kg. Then, hindlimb ischemia was induced using an orthodontic rubber band (Alpha Dental Equipment, Guangzhou, China) to ligate the left thigh above the trochanter. 4 h ischemia, and reperfused for 12 h. We provided postoperative analgesics: Buprenorphine (0.1 mg/kg body weight, every 8 h).

### 2.3 Drug treatments and experimental design

BBM was acquired from MCE (Shanghai; China). The dosage of BBM was determined based on prior research (Kathem et al., 2022; Qi et al., 2022). We selected 20 mg/kg for the low-dose group and 50 mg/kg for the high-dose group (Fan et al., 2022; Qi et al., 2022). A total of 48 mice (n = 12 per group) were randomly allocated into four groups: Sham group, IR group, IR + BBM (20 mg/kg) group, and IR + BBM (50 mg/kg) group. Mice in the IR and IR + BBM groups underwent hindlimb IR injury, while those in the Sham group did not receive any hindlimb IR injury. BBM was administered daily for 5 days before hindlimb IR injury (i.g.). Following the sacrifice of the mice, blood samples were obtained from the abdominal aorta. Muscle tissues were either frozen at −80 °C for additional analysis or preserved in an environmentally friendly GD muscle fixative solution for histological assessment.

### 2.4 Histological evaluation

Samples were immersed in environmental-friendly GD muscle fixative solution and stored at 4°C for 48 h. Transverse sections were stained with hematoxylin and eosin for inflammatory infiltration. Stained sections were captured by a bright-field microscope with a digital camera. The infiltrated inflammatory cells were identified, counted, and analyzed by an experimenter blinded to the grouping. The inflammatory cell count was analyzed by ImageJ software.

### 2.5 2,3,5-Triphenyltetrazolium Chloride (TTC) staining

After 12 h of reperfusion, mice were sacrificed for TTC staining. The gastrocnemius muscles were frozen for 1 min and sliced into transverse sections (1–2 mm thickness). The cut slices were cleaned with cold saline solution and subsequently incubated with TTC solution (Servicebio; Wuhan; China) at 37°C for 1 h.

### 2.6 Malondialdehyde assays

The levels of malondialdehyde (MDA) in gastrocnemius muscles were quantified using an MDA assay kit (Yuanye, Shanghai, China). In summary, the supernatant, obtained from the homogenization of muscle tissue, was combined with MDA assay reagent. This mixture was then heated at 95 °C f. Following the cooling process, the mixture underwent centrifugation at a temperature of 4°C. Subsequently, the absorbance of the resulting supernatants was measured at a wavelength of 535 nm. The concentration of MDA was reported as nanomoles per milligram of protein (nmol/mg protein).

### 2.7 TUNEL staining

The TUNEL assay is a technique employed to detect DNA fragmentation by marking the 3′-hydroxyl ends of double-stranded DNA breaks that occur during apoptosis. Following the preparation of tissues using paraffin embedding and paraformaldehyde fixation, staining was carried out with a Cell Apoptosis Detection Kit (Servicebio, Wuhan, China). The nuclei were made visible using DAPI. Representative areas were imaged using a laser scanning confocal microscope, and digital images were captured. The apoptotic index was determined as the average percentage of TUNEL-positive cells in each transverse section counted. The quantification of TUNEL-positive cells was performed using ImageJ software.

### 2.8 Immunofluorescence assays

For immunofluorescence, tissue sections underwent deparaffinized and heat-induced antigen retrieval in buffer solution. Prior to overnight incubation at 4 °C with the primary antibody, the tissue sections were blocked with 3% BSA for 1–2 h. Following this, the sections were washed three times with PBS. For immunofluorescence, the sections were incubated with F4/80 (Abcam, ab6640, 1:100), p65 (Proteintech, 10745-1-AP, 1:1000), 4-HNE (Abcam, ab48506, 1:100) or CD86 (Proteintech, 13395-1-AP, 1:500), fluor-conjugated secondary antibody for 1 h at room temperature. DNA was visualized with DAPI. Digital images of representative areas were captured with a laser scanning confocal microscope.

### 2.9 Western blot analysis

Mouse tissues were prepared and lysed. Protein concentration was determined by Bradford buffer. Total protein (20–40 µg/lane) was run in SDS–PAGE gels and transferred to nitrocellulose membranes. The target bands were detected with antibodies: NFE2L2 (Abcam, ab137550, 1:1000), GPX4 (Proteintech, 67763-1-Ig, 1:1000), Acsl4(Proteintech, 22401-1-AP, 1:1000), Nlrp3 (Thermo Fisher Scientific, 25N10E9, 1:500), Caspase-1(Santa Cruz Biotech, sc-56036, 1:200), Asc (Santa Cruz Biotech, sc-22514, 1:1000), cleaved caspase-3(Proteintech, 68773-1-Ig, 1:1000), cleaved PARP1(Hubio, SU0314, 1:1000), p65(Proteintech, 10745-1-AP, 1:1000), IKKα(Hubio, ET1611-15, 1:1000), IκBα(Hubio, ET1603-6, 1:1000), P-IκBα(Hubio, ET1609-78, 1:1000), anti-β-Actin (Bioworld, AP0060, 1:10,000); anti-Gapdh (Biodragon, B1030,; 1:1000), anti-Histone 3 (Abcam, ab1791, 1:2000). The blots were incubated with HRP-conjugated secondary antibodies (anti-rabbit or mouse) and visualized with enhanced chemiluminescence detection reagents. Relative changes in protein expression were estimated from the mean pixel density using ImageJ and normalized to Ponceau S, Histone 3 or β-Actin.

### 2.10 Quantitative real-time PCR

Total RNA was isolated from tissues or cells using RNA isolate Total RNA Extraction Reagent (Vazyme; Nanjing; China) according to the manufacturer’s instructions. qPCR analysis was performed with ChamQ Universal SYBR qPCR Master Mix (Vazyme; Nanjing; China) in Applied Biosystems 7300. β-actin expression was used as an endogenous control. The 2^−ΔΔCT^ method was used to analyze the relative fold changes. The primers were: forward 5′-GCC​ACT​GCC​GCA​TCC​TCT​TC-3′ and reverse 5′-AGC​CTC​AGG​GCA​TCG​GAA​CC-3′ for *β-Actin*; forward 5′-ATG​CTG​CTT​CGA​CAT​CTC​CT-3′ and reverse 5′-AAC​CAA​TGC​GAG​ATC​CTG​AC-3′ for *Nlpr3*; forward 5′-GAC​TCT​TGC​GTC​AAC​TTC​AAG​G-3′ and reverse 5′-CAG​GCT​GTC​TTT​TGT​CAA​CGA-3′ for *IL-1*β; forward 5′-ACG​GCA​TGG​ATC​TCA​AAG​AC-3′ and reverse 5′- GGT​CAC​TGT​CCC​AGC​ATC​TT -3′ for *Tnf-α*; forward 5′- AGT​TGC​CTT​CTT​GGG​ACT​GA -3′ and reverse 5′- GCC​ACT​CCT​TCT​GTG​ACT​CC -3′ for IL-6; forward 5′- ACC​TAC​CGC​ACC​CGA​GAT​G -3′ and reverse 5′- AAG​CCA​CTG​ACA​CTT​CGC​ACA -3′ for *iNOS*.

### 2.11 The calculation of the edema index

Muscle specimens were initially weighed to determine their wet weight and then underwent freeze-drying for 48 h to obtain the dry weight. The tissue edema index was calculated as the water content percentage using the formula: water content (%) = [(wet weight–dry weight)/wet weight] *100.

### 2.12 Statistics

All data are expressed as mean ± SEM. One-way ANOVA was conducted, followed by Tukey’s multiple comparisons test using GraphPad Prism 8.4.2. A P value of <0.05 was deemed significant. Results are presented as mean ± SEM. *p < 0.05, **p < 0.01, ***p < 0.001, ****p < 0.0001.

## 3 Results

### 3.1 Berbamine reduces IR-induced interstitial edema and muscle damage in mice

**FIGURE 1:**
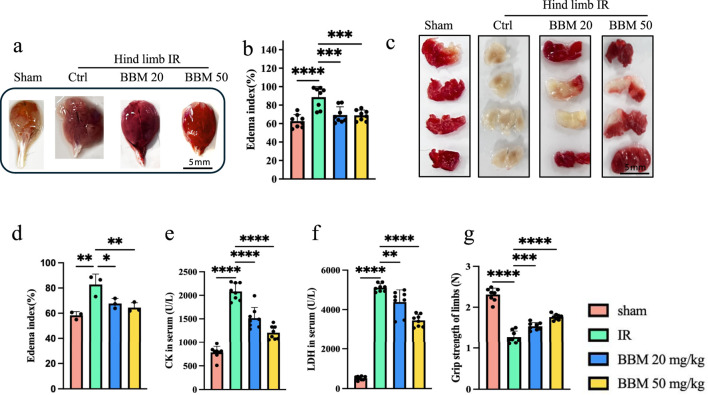
Berbamine treatment reduces interstitial edema and muscle damage in hindlimb IR injury mice. **(A)** Representative images of gastrocnemius muscles in mice with sham group, IR group, IR + 20 mg/kg BBM group, IR + 50 mg/kg group, n = 8 per group. **(B)** The edema index of gastrocnemius muscles, n = 3 per group. **(C, D)** The TTC staining and infarct size in gastrocnemius muscles of gastrocnemius muscles, n = 8 per group. **(E, F)** The serum LDH and CK levels, n = 8 per group. **(G)** The grip strength of limbs, n = 8 per group. p values are shown as the mean ± SEM, one-way analysis of variance (ANOVA) was performed. *p < 0.05, **p < 0.01, ***p < 0.001, ****p < 0.0001.

To determine whether BBM can protect mouse hindlimb muscles from IR injury, we first collected the hindlimb muscles of mice with IR injury, including those treated with BBM. Overall, we observed that the IR-induced interstitial edema in the IR group was markedly greater than in the IR + BBM group (Figures 1A, B). The infarct size of the gastrocnemius muscles was assessed using the TTC assay, which demonstrated that the infarct area in the IR + BBM groups was decreased compared to the IR group (Figures 1C, D). Furthermore, the serum levels of creatine kinase (CK) and lactate dehydrogenase (LDH) were higher in the IR group than in the BBM-treated groups, suggesting a higher level of tissue damage (Figures 1E, F). The grip strength of the mice’s limbs also indicated more pronounced muscle damage in the IR group (Figure 1G). In summary, BBM treatment (20 mg/kg and 50 mg/kg, intraperitoneal) administered 5 days prior to IR notably reduced, although did not eliminate, acute IR-induced injuries in the gastrocnemius muscles, including interstitial edema and muscle damage. Moreover, the efficacy of BBM increased with higher concentrations.

### 3.2 Berbamine inhibits IR-induced lipid ROS in mice

**FIGURE 2:**
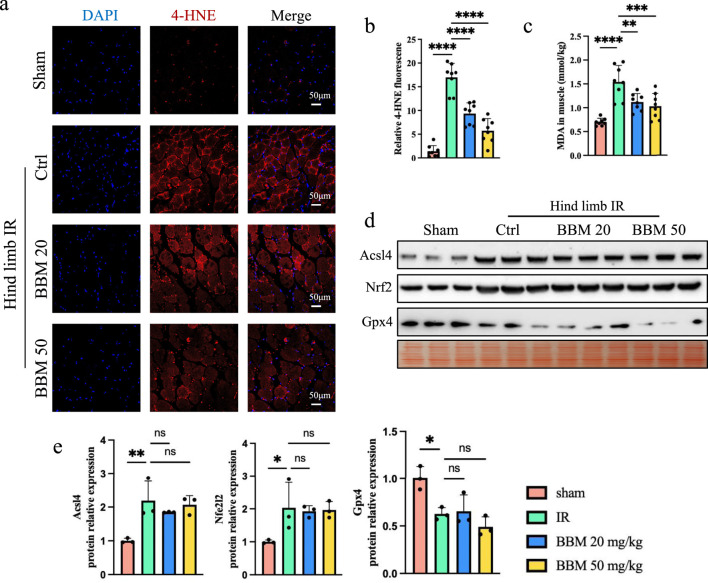
Berbamine inhibits lipid ROS in hind limb IR injury mice. **(A, B)** 4-HNE staining of gastrocnemius muscles in transverse sections. Lipid ROS were labeled with 4-HNE (red). Nuclei were labeled with DAPI (blue), n = 8 per group. **(C)** MDA levels in gastrocnemius muscles, n = 8 per group. **(D, E)** Representative immunoblot bands and quantification of Gpx4, Acsl4, and Nfe2l2 in gastrocnemius muscles, n = 3 per group. p values are shown as the mean ± SEM, one-way ANOVA was performed. *p < 0.05, **p < 0.01, ***p < 0.001, ****p < 0.0001.

Lipid ROS is the main molecular mechanism for oxidative damage to cell structures during organ ischemia-reperfusion and is also a key factor leading to cell death (Sakamaki et al., 1997; Zhang et al., 2014). Previous studies have found that BBM can directly scavenge DPPH prevent accumulation of lipid peroxides in living cells (Fan et al., 2022). To verify whether BBM can alleviate the increase in lipid ROS induced by reperfusion of ischemic tissue, we performed 4-HNE and detected the MDA levels in the tissue. 4-HNE staining indicate that, compared with sham treatment, hindlimb IR increased the level of lipid ROS, and pretreatment with different doses of BBM significantly reduced it (Figures 2A, B). In line with this, the MDA level was found to be lower in the IR + BBM groups compared to the IR group (Figure 2C). We analyzed proteins associated with lipid ROS generation pathways, including Glutathione peroxidase 4 (Gpx4), acyl-CoA synthetase long-chain family 4 (Acsl4), and nuclear factor erythroid-2 related factor 2 (Nfe2l2) (Figures 2D, E). Western blot analysis revealed that BBM did not affect Gpx4, Acsl4, and Nfe2l2. These findings suggest that BBM can suppress IR-induced oxidative stress in skeletal muscles and shield muscle tissues from damage caused by IR-induced lipid ROS.

### 3.3 Berbamine mitigates the level of inflammation induced by IR in mice

**FIGURE 3:**
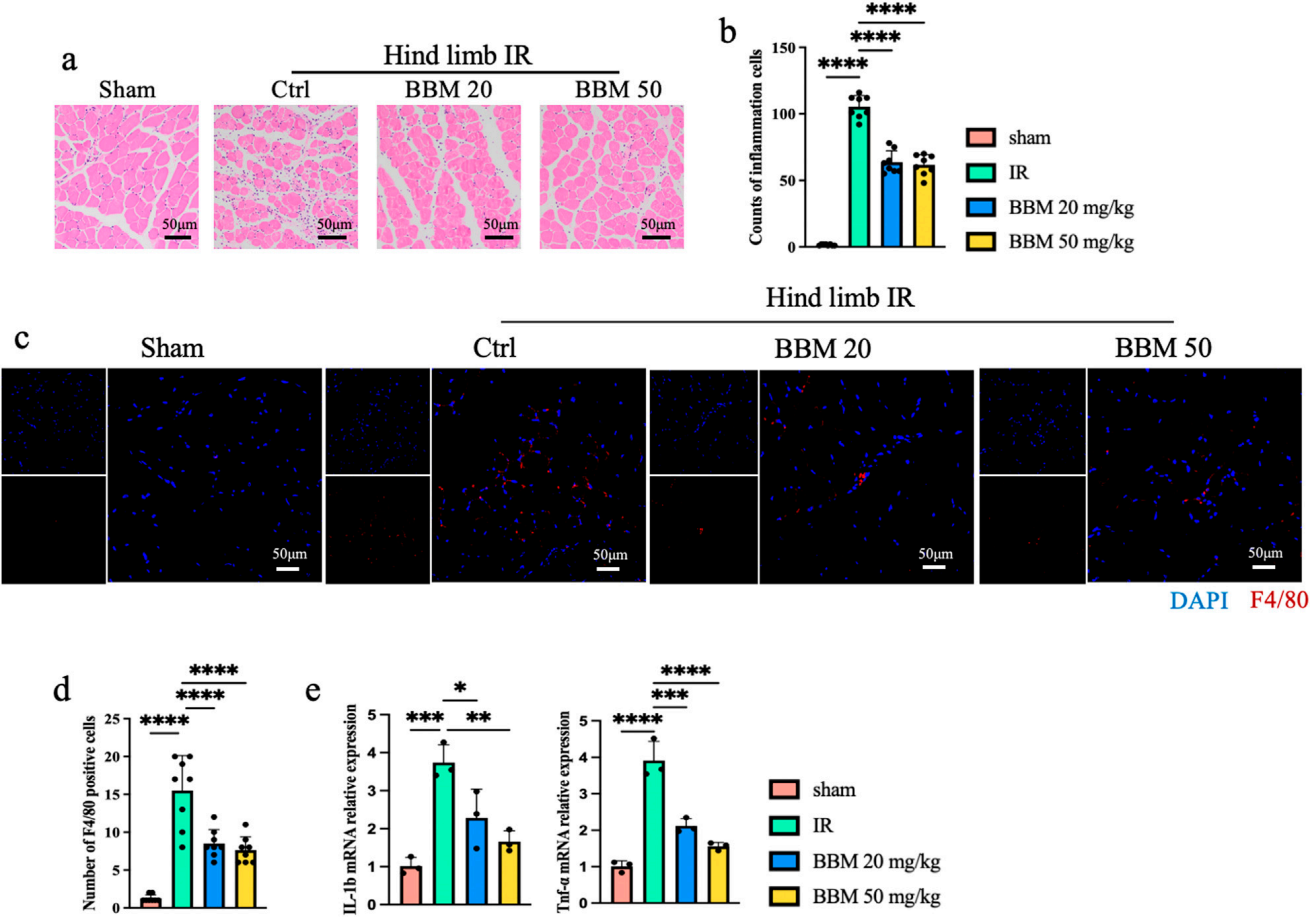
Berbamine Reduces the Level of inflammation in mice ischemic hind limb. **(A)** Representative Hematoxylin and Eosin staining (HE) images of gastrocnemius muscles in transverse sections, n = 8 per group. **(B)** Quantification of the number of inflammatory cells in Figure 3A, n = 8 per group. **(C, D)** Representative IF images and quantification of F4/80 (Red) gastrocnemius muscles in transverse sections, nuclei were labeled with DAPI (blue), n = 8 per group. **(E)** mRNA expression level of IL-1β and TNF-α in the gastrocnemius muscles of mice, detected by qPCR, n = 3 per group. p values are shown as the mean ± SEM, one-way ANOVA was performed. *p < 0.05, **p < 0.01, ***p < 0.001, ****p < 0.0001.

Inflammation is a crucial factor in exacerbating ischemia-reperfusion injury. To investigate the impact of BBM on IR-induced inflammation in mice, we conducted the following experiments. Hematoxylin and eosin (H&E) staining revealed significant infiltration of inflammatory cells in both the IR group and the IR + BBM groups, in contrast to the Sham group. Nevertheless, the administration of BBM led to a decrease in the number of inflammatory cells (Figures 3A, B) and reduced F4/80 staining (Figures 3C, D). Additionally, quantitative polymerase chain reaction (qPCR) results indicated that the expression of Interleukin-1β (IL-1β) and Tumor necrosis factor-α (TNF-α) in IR-affected muscle tissue was markedly elevated, and BBM was able to suppress the upregulation of IL-1β and TNF-α (Figure 3E). These experimental findings suggest that BBM can diminish tissue inflammation resulting from hind limb ischemia in mice.

### 3.4 Berbamine reduces IR-induced macrophage M1 polarization and activation of the Nod-like receptor protein-3(NLRP3) inflammasome in mice

**FIGURE 4:**
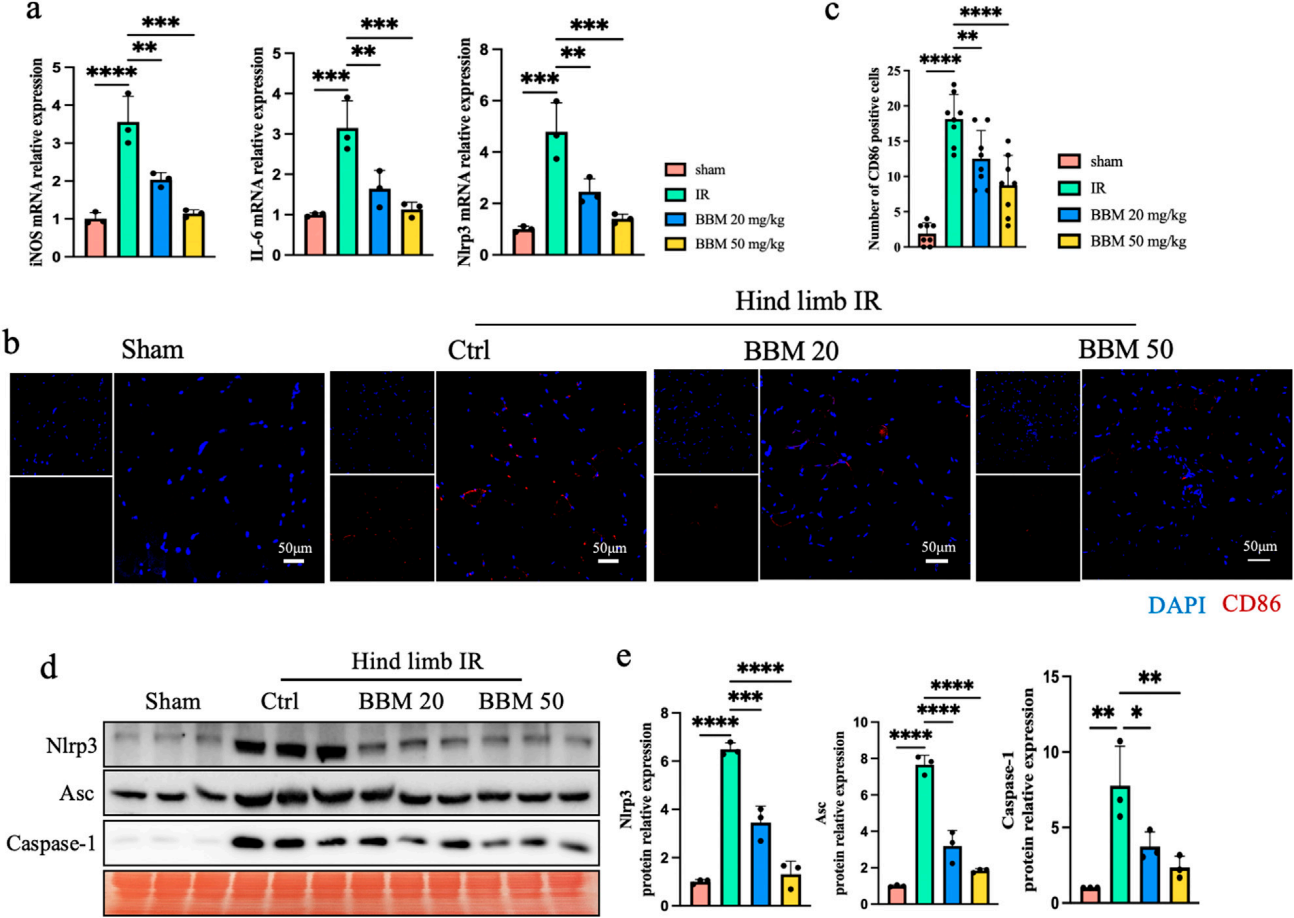
Berbamine Reduces IR-Induced macrophage M1 polarization and activation of the NLRP3 inflammasome in mice ischemic hind limb. **(A)** mRNA expression level of iNOS, IL-6 and NLRP3 in the gastrocnemius muscles of mice, detected by qPCR, n = 3 per group. **(B, C)** Representative immunofluorescence images and quantification of CD86 (red) in gastrocnemius muscles transverse sections, nuclei were labeled with DAPI (blue), n = 8 per group. **(D, E)** Representative immunoblot bands and quantification of Nlrp3, Asc, pro-Caspase-1 and cleaved-Caspase-1 in gastrocnemius muscles, n = 3 per group. p values are shown as the mean ± SEM, one-way ANOVA was performed. *p < 0.05, **p < 0.01, ***p < 0.001, ****p < 0.0001.

Macrophage M1 polarization promotes tissue inflammation and plays a significant role in ischemia-reperfusion injury (Li et al., 2024; Liu et al., 2021). We found through qPCR that BBM can inhibit the expression of macrophages M1 polarization marker, iNOS and IL6, induced by ischemia-reperfusion injury (Figure 4A). Furthermore, immunofluorescence revealed that macrophage CD86 staining was significantly increased in the ischemia-reperfusion tissue, and BBM attenuated the expression of CD86 (Figures 4B, C). Experimental data indicate that the rise in ROS levels during IR exacerbates tissue inflammation and activates the immune response through the NLRP3 inflammasome (Li et al., 2015; Zhao et al., 2022). Next, to assess the impact of BBM on NLRP3 inflammasome activation, we detected changes in the mRNA and protein expression levels of NLRP3 and related proteins using qPCR (Figure 4A) and Western blot (Figures 4D, F). As shown in the figure, the expression of Nlrp3, Apoptosis-associated speck-like protein containing CARD (Asc), and Caspase-1 were significantly increased after hindlimb IR at both the mRNA and protein levels. BBM pretreatment significantly decreased the upregulation of these proteins. Therefore, these results indicate that BBM can reduce the levels of IR-induced inflammation in mice, with the mechanism potentially involving BBM’s inhibition of macrophage M1 polarization and activation of the NLRP3 inflammasome.

### 3.5 Berbamine mitigates IR-induced apoptosis in mice

**FIGURE 5:**
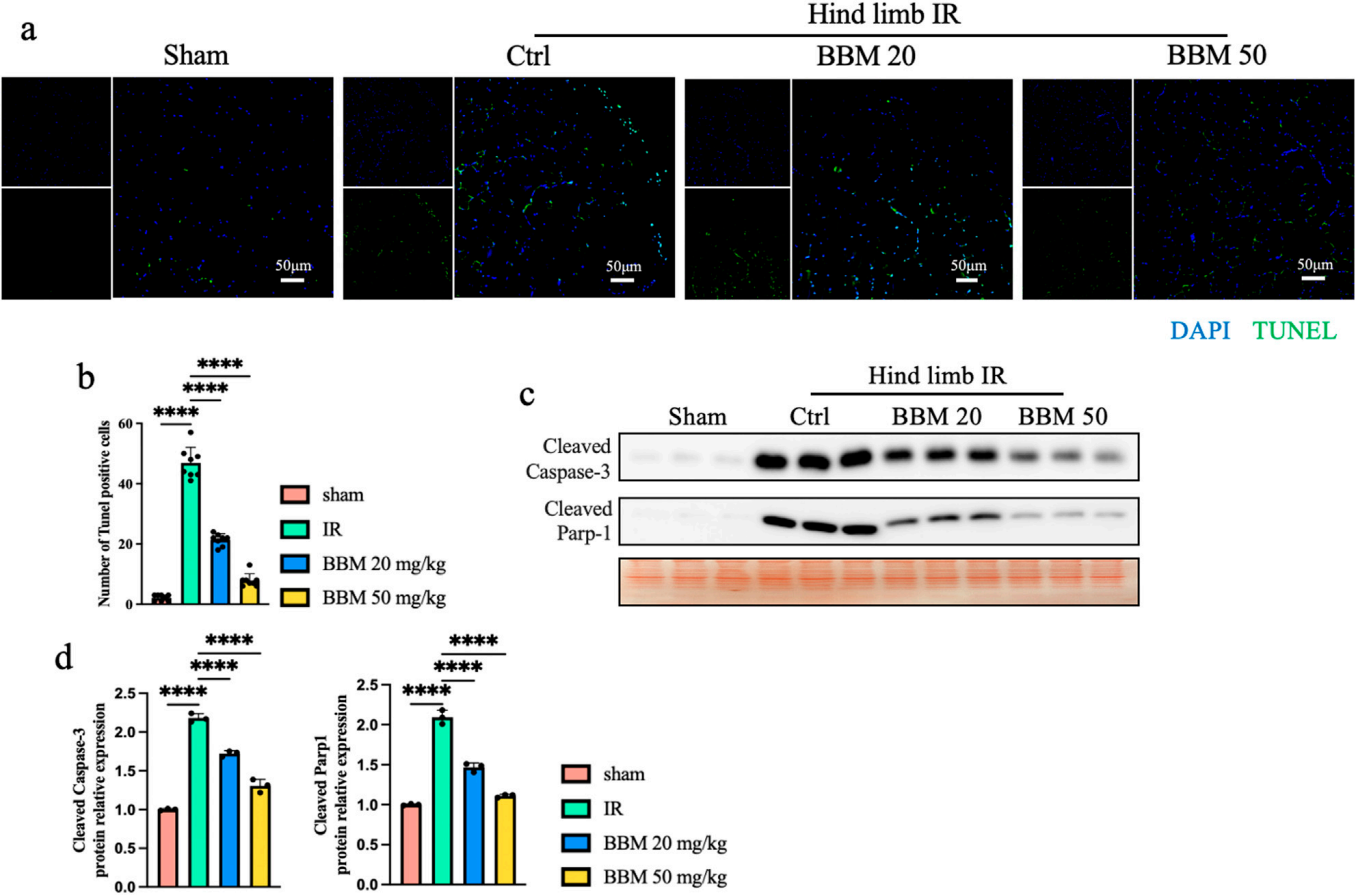
Berbamine reduces IR-induced apoptosis in mice ischemic hind limb. **(A, B)** Representative images of TUNEL staining (green) gastrocnemius muscles in transverse sections and quantitative statistical analysis showing the index of apoptotic cells, nuclei were labeled with DAPI (blue), n = 8 per group. **(C, D)** Representative immunoblot bands and quantitative analysis of cleaved-Caspase-3 and cleaved-Parp1 in gastrocnemius muscles. n = 3 per group. p values are shown as the mean ± SEM, one-way ANOVA was performed. *p < 0.05, **p < 0.01, ***p < 0.001, ****p < 0.0001.

Apoptosis is also triggered by IR. We initially employed the TUNEL assay to assess the impact of BBM on IR-induced apoptosis. The TUNEL assay revealed a higher number of apoptotic cells in both the IR group, whereas BBM pretreatment notably reduced the apoptosis index in comparison to vehicle treatment (Figures 5A, B). We also assessed the expression of several apoptosis-related proteins using Western blotting (Figures 5C, D). As depicted in the figure, we observed that following hindlimb IR, the expression of caspase-3 and cleaved poly ADP-ribose polymerase 1 (PARP1), which serve as proapoptotic proteins, was markedly increased. All these alterations in expression levels were significantly diminished upon pretreatment with BBM. In summary, our findings indicate that BBM can decrease cell apoptosis in muscles following hindlimb IR injury.

### 3.6 Berbamine inhibits P65 nuclear translocation

**FIGURE 6:**
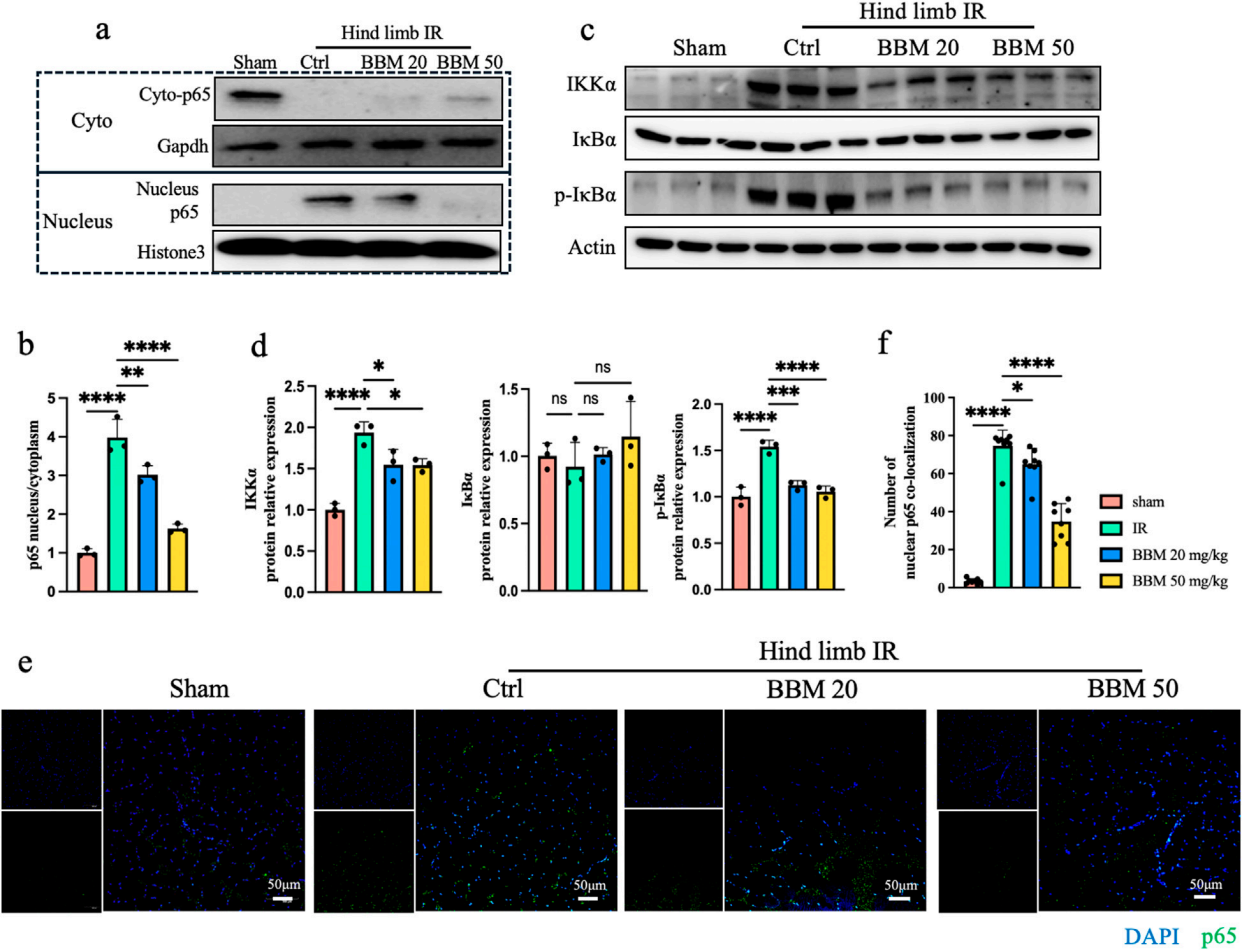
Berbamine inhibits p65 nuclear translocation. **(A, B)** Representative immunoblot bands and quantitative analysis of IKKα, IκBα, p-IκBα, A20 and p65 in gastrocnemius muscles, n = 3 per group. **(C, D)** Nuclear p65 protein expression in gastrocnemius muscles was analyzed by Western blot, n = 3 per group. **(E, F)** p65 nuclear translocation was determined by IF for p65 (green) and DAPI (blue) staining for DNA, n = 8 per group. p values are shown as the mean ± SEM, one-way ANOVA was performed. *p < 0.05, **p < 0.01, ***p < 0.001, ****p < 0.0001.

P65 is an important transcription factor for inflammatory factors and plays a role in promoting tissue damage during the occurrence of IR injury (Yu et al., 2020). Previous studies have found that BBM can downregulate phosphorylation of NF-kappa-B inhibitor alpha (IκBα), and subsequently inhibit p65 nuclear translocation, leading to decreased expression of the downstream targets of p65 (Liang et al., 2009). We first detected the expression of p65 in the mouse hindlimb ischemia-reperfusion model. Compared to the control group, the nuclear-to-cytoplasmic ratio of p65 expression in the IR mouse muscle tissue was elevated. Concurrently, BBM significantly suppressed the nuclear translocation of p65 in the IR condition (Figures 6A, B). To further investigate the inhibition of P65 nuclear translocation by BBM, we detected the phosphorylation level of IκB protein. We found that the expression of Inhibitory Kappa B Kinase α (IKKα) was significantly reduced in the BBM group, phosphorylated IκBα (p-IκBα) was almost undetectable, while the total IκBα remained almost unchanged (Figures 6C, D). These results indicate that BBM exerts its effects by downregulating the expression of p65.

## 4 Discussion

In this study, we found that BBM can inhibit ischemia-reperfusion-induced lower limb muscle injury by suppressing lipid ROS and P65 nuclear translocation. The main points to be underlined of this study can be summarized as follows: (i) The use of BBM can alleviate muscle tissue damage and muscle cell apoptosis; (ii) Administration of BBM can inhibit lipid ROS induced by muscle ischemia-reperfusion; (iii) BBM inhibits P65-mediated macrophage M1 phenotype polarization and muscle inflammation.

Hindlimb IR injury, frequently encountered in clinical settings during trauma and other emergencies, is a prevalent peripheral vascular condition (Apichartpiyakul et al., 2022). The pathogenesis of hindlimb IR injury is associated with cell apoptosis, oxidative stress, and inflammation (Küçük et al., 2021; Zhao et al., 2022; Apichartpiyakul et al., 2022). In this investigation, we demonstrated that the protective effect of BBM against hindlimb IR is attributed to its antioxidant and anti-inflammatory properties. The mechanisms at play may be intimately linked to the suppression of P65 activation and the capacity to eliminate lipid ROS.

Lipid ROS is closely related to ischemia-reperfusion injury (Bayır et al., 2024; Zhang et al.). During ischemia, anaerobic glycolysis occurs, producing lactic acid while disrupting calcium ion homeostasis. In the reperfusion phase, a large amount of oxygen and free radical-generating enzymes are reactivated, producing a surge of oxygen free radicals, such as superoxide anion (O2-) and hydroxyl radical (OH•), which attack the unsaturated fatty acids on the cell membrane, initiating lipid peroxidation. BBM belongs to the BBIQ compounds and can scavenge intracellular peroxides, effectively protecting mice from folic acid-induced renal tubular ferroptosis and acute kidney injury (Fan et al., 2022). In our study, we found that BBM can alleviate lower limb muscle injury caused by IR, and its mechanism is also related to the direct scavenging of lipid ROS. Meanwhile, BBM has no effect on the expression of GPX4 and ACSL4 but promotes the expression of NFE2L2. NFE2L2 is an antioxidant stress transcription factor, and in previous studies, we found that Oridonin can reduce muscle injury caused by ischemia-reperfusion by activating NFE2L2 and inhibiting the formation of the NLRP3 inflammasome (Zhao et al., 2022). This may also be one of the reasons why BBM protects the hind limb and alleviates IR injury.

Inflammation plays a crucial role in the process of ischemia-reperfusion injury, and its mechanism is related to the activation of P65 (Kuboki et al., 2009; Sung et al., 2002; Nishikawa et al., 2018). P65 is a core factor regulating the expression of inflammatory genes and plays a significant role in both innate and adaptive immune cells (Liu et al., 2017). P65 can promote macrophage M1 polarization and the release of pro-inflammatory factors (Wang et al., 2014),and it can also enhance the transcription of the NLRP3 gene and the formation of the NLRP3 inflammasome (Guo et al., 2015). BBM has been found to inhibit the activation of CaMKII γ and P65, as well as the nuclear translocation of P65 (Zhao et al., 2023; Gu et al., 2012; Liang et al., 2009). In this study, we found that BBM can inhibit the nuclear translocation of P65 caused by ischemia-reperfusion injury. Additionally, BBM can also suppress the increase in the expression of inflammatory molecules and the polarization of macrophages to the M1 phenotype, as well as reduce the infiltration of inflammatory cells caused by ischemia-reperfusion injury. While p65 signaling is beneficial for cell survival, in the setting of IR, the intense tissue inflammation triggered by the nuclear translocation of p65 signaling overshadows its positive effects. By inhibiting the inflammation in muscle tissue caused by IR injury, BBM can alleviate muscle tissue edema and muscle cell apoptosis, thus reducing tissue damage.

## 5 Conclusion

In summary, our findings suggest that BBM exerts a protective effect against hindlimb IR injury in mice. The underlying mechanism may be closely associated with the suppression of P65-mediated inflammation and the elimination of lipid ROS. Consequently, these results imply that the protective effect of BBM in the hindlimb IR model is substantial, and it may hold promise as a candidate for clinical trials as a therapeutic agent. Nevertheless, due to the discrepancies between animal models and the human body, particularly in terms of dosage, the current animal studies are not directly applicable to clinical settings. Additional clinical trials are essential to validate their efficacy and safety for human use.

## Data Availability

The original contributions presented in the study are included in the article/supplementary material, further inquiries can be directed to the corresponding author.

## Abbreviations

BBM, Berbamine; LLIRI, Lower limb ischemia-reperfusion injury; ROS, Reactive oxygen species; CaMKII γ, calmodulin-dependent protein kinase II γ; BBIQ, Bisbenzylisoquinoline; DPPH, 1,1-diphenyl-2-picryl-hydrazyl; CK, Creatine kinase; LDH, Lactate dehydrogenase; Gpx4, Glutathione peroxidase 4; Acsl4, acyl-CoA synthetase long-chain family 4; Nfe2l2, Nuclear factor erythroid-2 related factor 2; TNF-α, Tumor necrosis factor-α; IL-1β, Interleukin-1β; NLRP3, Nod-like receptor protein-3; ASC, Apoptosis-associated speck-like protein containing CARD; PARP1, poly ADP-ribose polymerase; IκBα, NF-kappa-B inhibitor alpha; IKKα, Inhibitory Kappa B Kinase α.
